# Influence of Different Light Spectra on Metabolism, Nutrition, and Photosynthetic Efficiency in the Development of Bell Pepper Seedlings

**DOI:** 10.1021/acsomega.6c03839

**Published:** 2026-08-15

**Authors:** Vanessa Susana Rech Bisi, Henrique Poersch, Ketelyn Rubert, Ruan Fogaça Feijó, Wendel Paulo Silvestre, Gabriel Fernandes Pauletti

**Affiliations:** † Postgraduate Program in Process Engineering and Technologies (PGEPROTEC), 58802 University of Caxias do Sul, Caxias do Sul, Rio Grande do Sul 95070-560, Brazil; ‡ 2 Course of Agronomy, 58802 University of Caxias do Sul, Caxias do Sul, Rio Grande do Sul 95070-560, Brazil

## Abstract

The spectral quality of light plays a fundamental role in regulating plant growth, photosynthesis, and metabolism, with LED technology being a promising tool for optimizing seedling production in controlled environments. However, studies that comprehensively evaluate the effects of different light spectra on the biometric, physiological, biochemical, and nutritional responses of pepper (*Capsicum annuum L*.) seedlings are still limited. Thus, this study aimed to evaluate the effect of different LED light spectra. This study investigated the effects of the colors white light (W), full-spectrum white light (WF), blue (B – 450 nm), red (R – 660 nm), 50% red (660 nm) and 50% blue (450 nm) (RB), and green (G – 530 nm) on the growth, photosynthetic performance, production of bioactive compounds, and nutrient absorption in bell pepper seedlings. The experimental design was completely randomized, with three replicates of 10 plants per treatment. The results were subjected to analysis of variance (ANOVA), and the parameters with statistical significance were analyzed using Tukey’s multiple comparison test with a 95% confidence interval. Biometric parameters, photosynthetic parameters, chlorophyll fluorescence, phenolic compounds, flavonoids, anthocyanins, and macro- and micronutrient contents were evaluated, with the results integrated using principal component analysis (PCA). The different light spectra promoted specific and complementary physiological responses. The results indicated that R light promoted greater plant height and accumulation of aboveground biomass, while W light and the RB combination favored leaf expansion, and RB light resulted in greater absorption of essential micronutrients such as Zn, Cu, and Mn. The G spectrum induced greater accumulation of total phenolic compounds, suggesting the activation of secondary metabolic pathways related to oxidative stress. Photosynthetic performance was optimized under W light, reflecting greater CO_2_ assimilation. B light showed less expressive results for biometric parameters, photosynthetic assimilation rate, and water use efficiency. Principal component analysis explained 80.46% of the total variability of the data, highlighting associations between the different light spectra and the biometric, physiological, biochemical, and nutritional responses of the seedlings. The results demonstrated that the spectral quality of light can modulate, in an integrated way, the growth, photosynthetic performance, mineral nutrition, and secondary metabolism of pepper seedlings, according to the production objective. These findings reinforce the potential of LED technology as a tool to optimize the quality of seedlings produced in controlled environments.

## Graphical abstract

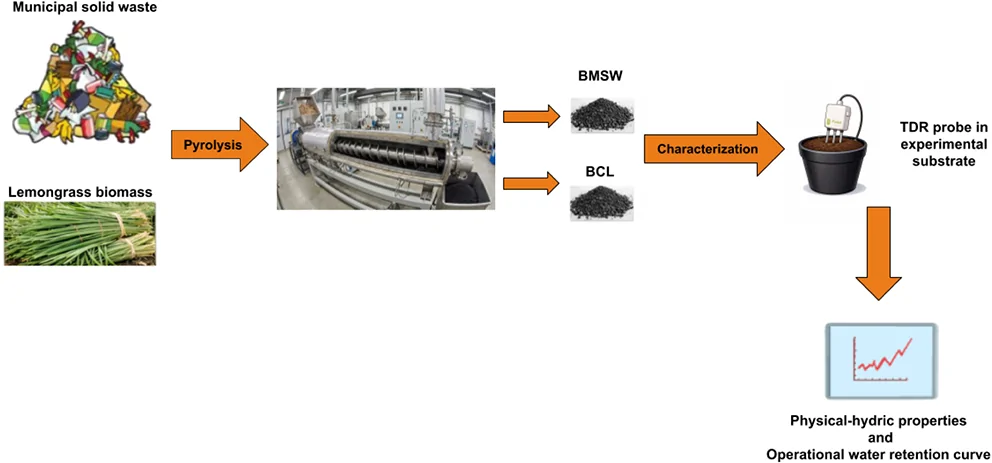

## Introduction

Bell pepper (*Capsicum annuum L*.) cultivation is among the main vegetables grown worldwide, showing wide adaptation to different edaphoclimatic conditions and production systems, including open-field cultivation and protected environments. In addition to its nutritional importance, the species has high agronomic value due to the significant demand for high-quality fruits and the need for efficient and sustainable production systems,^9^ since it can be cultivated in different regions and production systems. The production chain involves both family farmers and technologically advanced producers, contributing to the diversification of horticultural production and the supply of the consumer market.^25^

Commercial bell pepper production is predominantly based on the use of seedlings produced in nurseries or protected environments before the final transplanting. This practice allows for greater control of environmental factors during germination and initial growth, favoring the production of more uniform, vigorous seedlings with better phytosanitary quality, essential characteristics for the proper establishment of plants in the field and for the productive performance of the crop.^34,35^

In this context, obtaining uniform and vigorous seedlings is a fundamental step for successful cultivation, since it directly influences the initial establishment of the plants, the efficiency in the use of production resources, and the uniformity of development throughout the cycle. In vegetable crops with a relatively short cycle, such as bell peppers, seedling quality is considered one of the main factors associated with the agronomic and productive performance of the crop.^22^

With the advancement of agricultural technologies focused on seedling production, new possibilities are emerging to improve plant performance from the initial stages such as indoor cultivation systems with artificial lighting using LEDs (light-emitting diodes). This cultivation method represents a promising alternative for production in greenhouses and indoor environments, especially in urban agriculture, as it allows precise control of environmental parameters such as temperature, humidity, photoperiod, light intensity, and quality, enabling production at different times of the year, regardless of climatic conditions.^14,32^

Furthermore, the spectral control provided by LEDs allows the use of monochromatic and polychromatic spectra with reduced heat emission, favoring the positioning of luminaires close to plants and increasing lighting efficiency.^24^ The energy efficiency of this technology stems from the lower energy demand for generating luminous flux, the reduced thermal emission, and the high lifespan of the equipment, which can vary between 20 and 50 thousand hours,^16^ and can also be associated with sensors and controllers for crop monitoring.^32^

The widespread use of LEDs in horticulture is related to the possibility of employing different light spectra throughout the phenological stages of plants, favoring photosynthetic efficiency, vegetative growth, and standardization of production.^14^ This occurs because the spectral quality of light exerts a determining influence on physiological, morphological, and metabolic processes, being perceived by a specialized set of photoreceptors, including phytochromes, cryptochromes, and phototropins.^39^ These systems allow the integration of distinct light signals, promoting fine adjustments in plant growth and metabolism in response to the light environment.^39,40^

In addition to regulating photosynthesis, the spectral quality of light influences the mineral absorption and secondary metabolism in plants. Recent evidence indicates that different wavelengths modulate the expression of nutrient transporters and signaling between shoots and roots, affecting the efficiency of absorption and utilization of macronutrients, especially under combinations of blue and red light.^12,46^

In parallel, light regulates the biosynthesis of phenolic compounds through the activation of the phenylpropanoid pathway, mediated by photoreceptors, such as phytochromes and cryptochromes. This process involves the induction of key enzymes, such as phenylalanine ammonia-lyase (PAL), resulting in a greater accumulation of total phenolics, frequently associated with antioxidant responses and protection against stress.^36,37^ Complementarily, flavonoids are strongly influenced by light quality, especially blue light, which activates cryptochromes and regulates genes of the phenylpropanoid pathway, promoting an increase in flavonols and anthocyanins and contributing to photoprotection and the antioxidant capacity of plants.^37,38^

In this context, different wavelengths were used experimentally to elucidate their specific functions. Red light (R), predominantly perceived by phytochromes, regulates processes such as germination, leaf expansion, and plant architecture through the interconversion between the active and inactive forms of the pigment, being strongly associated with photosynthetic efficiency and growth control.^41^

In contrast, blue light (B), perceived by cryptochromes and phototropins, plays a central role in stomatal regulation, photomorphogenesis, and the activation of metabolic pathways related to the synthesis of phenolic compounds and flavonoids, which are important for photoprotection.^42^ The combination of red and blue light (RB) is often considered more efficient for plant growth, as it integrates complementary signals from different photoreceptors, promoting a balance between photosynthesis, morphological development, and secondary metabolism.^43^

On the other hand, green light (G) has a high penetration capacity in the leaf canopy, reaching deeper layers of plant tissues and contributing to the redistribution of light energy, in addition to modulating physiological responses associated with shading and the plant’s energy balance.^44,45^ Treatments with white light (W) and full white light (WF) simulate spectra closer to solar radiation, allowing the simultaneous activation of multiple photoreceptors and functioning as comparative standards to evaluate the isolated and combined effects of different spectral radiations.

Optimizing the light source is fundamental in production systems that use artificial lighting, since light provides the energy necessary to produce photoassimilates,^24^ which support plant growth and development.^7^ The selective emission of wavelengths by LEDs, mainly in the B (∼450 nm) and R (∼660 nm) ranges, coincides with the main absorption peaks of chlorophylls A and B.^14^ However, the efficiency of the lighting system depends not only on the spectrum used but also on the cultivated species, since different plants may present distinct physiological responses.^18^

This variability was observed by^26^ when evaluating supplementation with pink artificial light (blue: 430 nm and red: 670 nm) in lettuce and cauliflower seedlings. The authors found superior biochemical responses in lettuce under light supplementation compared to natural light, while for cauliflower, no significant differences were observed, except for the levels of total phenolic compounds. Similarly,^13^ it was found that cucumber (*Cucumis sativus*) seedlings subjected to a higher ratio of R light than blue light showed better biometric results, higher chlorophyll content, and a higher net photosynthetic rate.

The influence of spectral composition was also demonstrated by Trojak et al.,^31^ who evaluated the partial replacement of R light by G light in tomato seedlings. The authors observed that the inclusion of 30% G light in combinations containing B and R light promoted beneficial effects under low-light conditions, while supplementation with 40% G light reduced dry biomass production. Furthermore, changes in the ratio between G light and R light modified the growth pattern and biomass accumulation of the plants.

Complementary results were obtained by Gao et al.,^8^ who supplemented tomato seedlings grown under continuous R light (650 nm; 220 μmol m^–2^ s^–1^) with B light (450 nm) at different intensities and frequencies. The authors found that the application of high-intensity, short-duration B light promoted greater shoot biomass production, greater enzymatic activity, and greater carbohydrate accumulation when compared to low-intensity, long-duration supplementation, considering the same daily light integral (DLI).

Similarly,^20^ light supplementation was evaluated in basil, red cabbage, and smooth mustard seedlings using red light (87.5% red and 12.5% blue) and W light (40% red, 10% blue, and 50% green). The authors observed improvements in the productive parameters of the seedlings without significant changes in the levels of phenolic compounds and flavonoids, indicating that light supplementation can optimize production without compromising the quality of the secondary metabolites evaluated.

Despite the frequently reported positive results for the R and B spectra, the observed effects are not consistent across species. While some studies report increased biomass and photosynthetic activity, others find limited responses or even reduced growth, suggesting a strong influence of genotype, light intensity, and the spectral ratio used. Furthermore, the discrepancies observed in the literature may be related to differences in photosynthetic flux density, total daily light (TDL), the phenological stage evaluated, and intrinsic characteristics of each plant species.

Although several studies have evaluated the effects of LED lighting on horticultural crops, most investigations focus on isolated parameters, such as growth, photosynthesis, or the production of secondary metabolites. For pepper seedlings, studies that simultaneously integrate biometric, nutritional, photosynthetic, and biochemical responses under different spectral compositions are scarce. This integrated approach is important for understanding the physiological mechanisms involved in the formation of high-quality seedlings in indoor cultivation systems.

In this sense, the spectra selected for this study were chosen because they represent different mechanisms of light perception in plants. R light is associated with the activation of phytochromes and vegetative growth; B light acts mainly through cryptochromes and phototropins, regulating stomatal opening and photomorphogenesis; G light has a greater capacity for penetration into the plant canopy; while W and WF lights simulate conditions closer to solar radiation. The RB combination was included because it represents one of the most widely used spectral compositions in commercial indoor growing systems.

Therefore, the objective of this study was to evaluate the effects of applying different light spectra emitted by LEDs on the initial production of bell pepper seedlings, considering the biometric, nutritional, physiological, photosynthetic, and biochemical parameters. To this end, it was hypothesized that spectra containing R and B radiation promote greater growth and photosynthetic performance of seedlings, while G light favors mechanisms related to secondary metabolism and the accumulation of phenolic compounds.

## Materials and Methods

The experiment was conducted in a climate-controlled growing room, containing shelves arranged with different LED lights, next to the Plant Environment Studies Laboratory (LESPA) at the University of Caxias do Sul (UCS). The temperature of the growing room was kept constant at 25 ± 2 °C, and the relative humidity at 75 ± 5%. The photosynthetic photon flux density (PPFD) was 180 μmol·m^–2^ ·s^–1^, which was verified periodically during the experiment to ensure that the target value was maintained and the PPFD remained uniform among the treatments. It was applied a photoperiod of 12 h (light:dark), corresponding to a DLI of 7.8 mol·m^–2^ ·day^–1^. These values were adapted from literature data^33,43,47^ and studies carried out by the research group,^20,26^ aiming to provide an amount of photosynthetically active radiation capable of sustaining seedling growth throughout the experimental period.

Six treatments were performed according to the wavelength–frequency of each light, namely: in the colors white light (W), full-spectrum white light (WF), blue (B – 450 nm), red (R – 660 nm), 50% red (660 nm) and 50% blue (450 nm) (RB), and green (G – 530 nm). The graphs of the relative spectral radiance as a function of wavelength for each of the lighting types used are presented in Figure 1.

**Figure 1:**
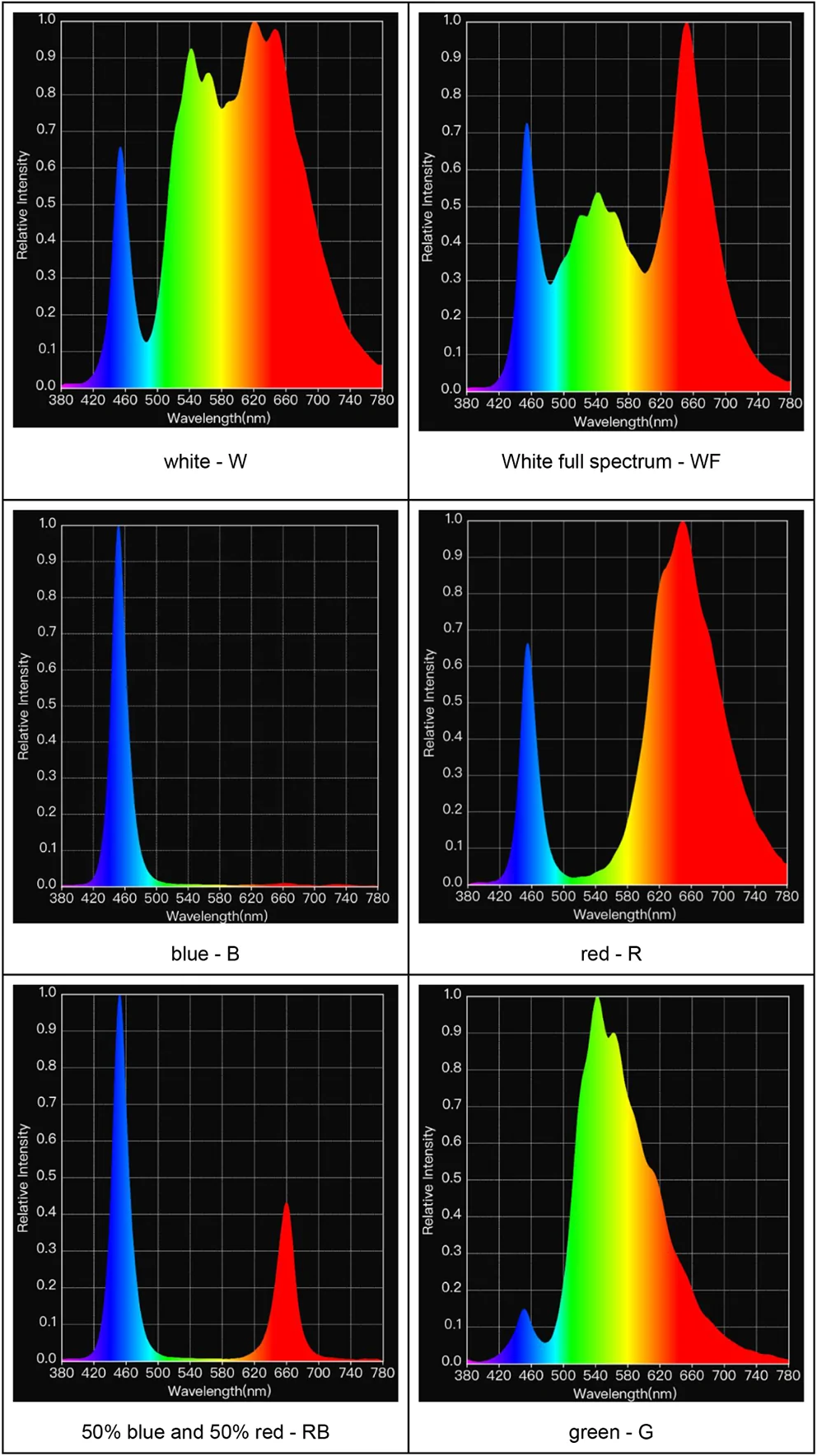
Graphs of relative spectral radiance for the wavelength range of 380–780 nm of the different types of LED light used in the experiment were obtained using an Asensetek model ALP-01 spectroradiometer.

The light treatments consisted of modules based on LEDs from the Lumileds LUXEON SunPlus 2835 family, combining Cool White, Lime, Royal Blue, Purple, Purple and Lime, Deep Red, and Royal Blue emitters, according to each experimental treatment. The spectral characterization presented in this study was based on the nominal specifications provided by the manufacturer, determined under standardized operating conditions, with a junction temperature (Tj) of 25 °C and a forward current (If) of 120 mA, as described in the LED technical documentation. It should be noted that all treatments were standardized to the same photosynthetic photon flux density (PPFD) at the canopy level, corresponding to 180 μmol m^–2^ s^–1^. Thus, the effective light intensity received by the seedlings remained identical between treatments, with only the spectral composition varying according to each LED source within the PAR range. The characteristics of the experimental emission spectra shown in Figure 1 are presented in Table 1.

**Table 1:** Spectral Characteristics of the LED Lighting Treatments Used in the Experiment^a^

Treatment	Photonic efficiency (μmol J^–1^)	Blue (400–500 nm) (%)	Green (500–600 nm) (%)	Red (600–700 nm) (%)	Far red (700–800 nm) (%)	R:B relation
W	2.14	9.50	39.67	50.83	9.00	5.35
WF	1.73	24.60	41.32	34.08	5.87	1.39
G	2.22	4.29	68.05	27.66	2.33	6.45
R	2.05	15.45	7.23	77.32	16.62	5.00
B	2.06	99.33	0.52	0.60	0.23	0.00
RB	2.52	47.13	0.27	52.59	0.24	1.12

a Data were obtained from the technical specifications provided by the manufacturer Lumileds.

Pepper seedlings (commercial variety Dallyla from Syngenta) were grown in plastic trays with commercial substrate Carolina Soil (Brazil) composed of sphagnum peat, expanded vermiculite, and carbonized rice husk. The product has a pH of 5.5 ± 0.5 and an electrical conductivity of 0.7 ± 0.3 mS cm^–1^. Its approximate density is 145 kg m^–3^, with a water retention capacity of around 55%. The substrate is characterized by high aeration, low density, and good water retention, being indicated for seedling production, seed germination, and rooting of cuttings, according to the manufacturer’s instructions.

The experimental design used was completely randomized, with three replicates per treatment. The experimental unit consisted of 10 plants, totaling 30 plants per treatment. For statistical analyses, the average of the 10 plants (replicates) from each experimental unit was used. The number of replicates adopted is similar to that used in studies involving the physiological evaluation of seedlings under LED lighting.

To minimize positional effects, the trays containing the experimental triplicates were distributed homogeneously in the central position of the shelf, which measured 1.00 m x 0.50 m, 0.30 m away from the LEDs, and each experimental unit occupied an independent position within the cultivation system. Each shelf was equipped with two LED bars installed in parallel and distributed along its length, providing uniform illumination over the entire cultivation area; for this reason, the positions of the trays were not alternated throughout the experiment.

After seed germination, the seedlings received a nutrient solution, prepared according to Hoagland et al.,^11^ whose composition remained unchanged throughout the experimental period. The total cultivation period was 33 days from sowing to harvest, corresponding to the seedling stage prior to transplanting, during which biometric, nutritional, photosynthetic, and biochemical parameters were evaluated. During the experiment, environmental conditions remained relatively stable. The minimum recorded temperature was 19 °C, while the maximum reached 27 °C, resulting in an average temperature of 25 °C and a thermal amplitude of 8 °C. The relative humidity varied from 52% to 72%, with an average value of 65%. During the experiment, the environmental conditions remained stable, without important variations in temperature and relative humidity that could influence the plants’ growth.

The biometric analyses were performed 33 days after sowing (DAS), corresponding to the stage at which the seedlings were considered to be suitable for transplanting. Photosynthetic measurements and chlorophyll fluorescence analyses were performed between 28 and 30 DAS using fully expanded intermediate leaves.

Biochemical and nutritional analyses were performed at the end of the growing period (33 DAS). The evaluation period was selected because it corresponds to the final stage of seedling development, which is commonly used for commercial transplanting.

Root length, shoot length, and internode distance were obtained by direct measurement using a millimeter ruler. To determine the dry biomass of the shoots and roots, the plant material was dried in a forced-air oven (DeLeo, Brazil) for 48 h at a temperature of 35–40 °C. The levels of macronutrients (N, P, K, Ca, Mg, and S) and micronutrients (Cu, Zn, Mn, Fe, and B) present in the plant material were determined according to the methods described by Malavolta et al.^19^ at the Soil Chemistry and Fertility Laboratory of the University of Caxias do Sul (LQFS/UCS).

Leaf area was measured using the Android operating system application, Easy Leaf Area, a tool that uses a mobile device’s camera to automatically measure the green area of leaves from photos. For measurement, a 4.0 cm^2^ red paper was placed next to the plants for color calibration, as described by Easlon et al.^6^

Photosynthetic parameters related to transpiration rate (E), stomatal conductance (g), photosynthetic assimilation rate (A), and water use efficiency (WUE) were evaluated using a portable infrared gas analyzer (IRGA, ADC Bioscientific, model LCP-SD) on a fully expanded intermediate leaf. Photosynthetic measurements were performed between 9:00 and 11:00 AM, 30 days after sowing (DAS). Measurements were conducted under ambient CO_2_ concentration (∼400 μmol mol^–1^), without external CO_2_ injection, using photosynthetically active radiation (PAR) corresponding to each light treatment. The temperature of the leaf chamber was maintained at 25 ± 2 °C.

Instantaneous chlorophyll fluorescence (FT) and photosystem II quantum efficiency (QY) were determined in the presence and absence of light, respectively, through direct measurement of the leaves by using a portable fluorometer (FluorPen).

Total chlorophyll content was obtained using the methodology described by Ross,^27^ and for the determination of total flavonoid content and total phenolic compound content, the methodology described in ref. 20 was used.

Each experimental triplicate constituted a sampling unit for physicochemical analyses. Additionally, to ensure precision, accuracy, and reliability of the results, all determinations were performed using an analytical blank and verified by means of calibration standards prepared according to the analytical methodologies employed.

The results obtained were evaluated for homogeneity (Levene’s test) and normality of residuals (Shapiro-Wilk test) and then subjected to analysis of variance (ANOVA). For all variables analyzed, the assumptions of homoscedasticity and normality of residuals were met, validating the use of ANOVA. Parameters that showed statistical significance were analyzed using Tukey’s multiple comparison test with a 95% confidence interval (α = 0.05). Statistical analyses were performed using AgroEstat software (Brazil).

Principal component analysis (PCA) was performed to evaluate, in an integrated manner, the biometric, photosynthetic, biochemical, and nutritional responses of pepper seedlings subjected to different LED light spectra. The analysis was conducted using individual data from all experimental replicates, including biometric variables (plant height, stem diameter, and total dry mass), physiological variables (net CO_2_ assimilation rate: *A*, stomatal conductance: *gs*, transpiration rate: *E*, and maximum quantum efficiency of photosystem II: Fv/Fm), biochemical variables (total phenolic compounds, total flavonoids, and total anthocyanins), and nutritional variables (macro- and micronutrient content in plant tissue).

Due to the different measurement scales of the variables, the data were previously standardized (mean = 0 and standard deviation = 1), and the PCA was performed based on the correlation matrix between the variables. The interpretation of the principal components considered the percentage of variance explained by each component and the loading coefficients of the variables, allowing identification of the main factors responsible for the discrimination of the treatments and the associations between the evaluated variables. The analyses were performed using Origin Pro software, version 2026 (OriginLab Corporation, Northampton, MA, USA).

## Results and Discussion

The biometric parameters evaluated showed a statistically significant difference between the treatments, except for root length, as demonstrated in Table 2.

**Table 2:** Biometric Parameters of Pepper Seedlings Grown under Different Light Spectra

Treatment	Leaf area (cm^2^)	Distance between nodes (cm)	Height (cm)	Root length^a^ (cm)	Aboveground dry biomass (g)	Root dry biomass (g)
W	156.18 ± 10.64 a	3.63 ± 0.25 a	13.45 ± 1.32 b	11.60 ± 2.42	0.37 ± 0.07 ab	0.15 ± 0.05 bc
WF	143.30 ± 16.98 ab	2.25 ± 0.29 b	11.00 ± 0.70 c	11.25 ± 1.87	0.30 ± 0.08 ab	0.25 ± 0.09 a
B	95.76 ± 12.26 bc	1.25 ± 0.29 c	9.10 ± 0.97 d	12.25 ± 3.34	0.19 ± 0.06 c	0.11 ± 0.04 c
R	141.07 ± 26.68 ab	3.25 ± 0.29 a	15.50 ± 1.35 a	10.10 ± 2.27	0.38 ± 0.096 a	0.19 ± 0.06 ab
RB	156.44 ± 22.45 a	1.20 ± 0.29 c	7.80 ± 0.34 e	10.70 ± 1.51	0.27 ± 0.06 bc	0.19 ± 0.07 ab
G	80.89 ± 32.11 c	2.25 ± 0.29 b	12.05 ± 0.69 c	11.40 ± 1.12	0.36 ± 0.09 ab	0.14 ± 0.05 bc
p-value	0.0032	<0.0001	<0.0001	0.3545	<0.0001	<0.0001
Coefficient of variation (%)	16.74	12.22	8.41	19.69	24.83	34.91

a Not significant by ANOVA at 5% probability of error. Values presented as mean ± standard deviation. White light (W), full-spectrum white light (WF), blue (B – 450 nm), red (R – 660 nm), 50% red (660 nm) and 50% blue (450 nm) (RB), and green (G – 530 nm).

For leaf area, the W, WF, R, and RB spectra showed the largest dimensions, respectively: 156.18 cm^2^, 143.30 cm^2^, 141.07 cm^2^, and 156.44 cm^2^. The white light (W) and the red and blue combination (RB) spectra favored leaf expansion, being superior to the B and G spectra, which showed less leaf area expansion, 95.76 cm^2^ and 80.89 cm^2^, respectively, indicating that these spectra tend to compact the leaves. It should be noted that, in relation to the G spectrum, the W and RB light promoted a 93% increase in leaf area.

The study carried out by Snowden et al.^29^ on the effects of blue light (400–500 nm) found that pepper plants subjected to this spectrum showed a reduction in leaf area index when compared to the red (600–700 nm) and green (500–600 nm) spectra, suggesting that at longer wavelengths, canopy closure may be favored. This trend was also observed in this work for the B-spectrum.

For Nie et al.,^23^ light G (λ_max_ = 520 nm) showed an increase in leaf area in pepper plants, indicating potential for plant growth and development. In this study, however, it was possible to observe that this spectrum presented one of the lowest leaf area results, but it is aligned with the leaf reduction presented by light B (λ_max_ = 460 nm) in the study of the aforementioned authors.

The internode distance allowed us to observe that plants subjected to W and R light spectra showed greater elongation, with 3.63 and 3.25 cm, respectively. The greater spacing in internodes may be characteristic of the action of R light, which keeps phytochrome active and stimulates cell elongation. When the spectrum with the RB combination was used, more compact plants were observed (1.20 cm), as the mixture of lights helped regulate the hormonal balance, resulting in more balanced growth,^4^ avoiding etiolation.

The tendency for internode reduction with the use of the RB spectrum combination was also verified by Lin et al.,^17^ where they employed B light (∼453 nm) and R light (∼660 nm) in a 10:8 ratio. The simultaneous presence of B light probably restricted cell expansion, helping to reduce stem elongation.

When evaluating internodes in pumpkin cultivation,^17^ it was also observed that there was better development under red light (660 nm). However, they observed that blue light (453 nm) also showed a positive response, unlike what occurred with bell peppers in this experiment, where a shorter internode length (1.25 cm) was observed. This fact is consistent with the study by Rizzon et al.,^26^ highlighting the difference in physiological response between species.

These results reinforce the idea that the effects of light spectra on plant elongation do not present a standard effect. The same spectral range can promote contrasting responses between species because of differences in photoreceptor sensitivity, hormonal regulation, and the ability to acclimate to the light environment. Therefore, extrapolation between crops should be made with caution.

Considering the results obtained for plant height, it was observed that the R spectrum showed the greatest height (15.50 cm), in accordance with the trend of shoot elongation.^41^ This value corresponds to a 99% increase in plant height compared to the RB treatment, which showed the lowest height. This same trend was observed by refs. 17 and 23 at wavelengths close to 660 nm. The colors W, WF, and G showed plants with intermediate growth, with heights of 13.45, 11.0, and 12.05 cm, respectively.

The shorter heights in treatments RB (7.80 cm) and B (9.1 cm) may be associated with the lower photosynthetic assimilation rate promoted by these spectra and the effect of internode shortening. Color R also showed higher shoot biomass (0.38 g), a behavior similar to that observed by Nie et al.^23^ Spectra W (0.37 g), WF (0.30 g), and G (0.36 g) did not differ statistically from R. The B spectrum showed the lowest biomass (0.19 g).

Root length did not show a significant difference between treatments, which may be associated with growth limitations imposed by the growing container; however, root biomass showed a significant variation.

The WF spectrum showed the highest accumulation of root biomass (0.25 g), followed by those of R and RB (0.19 g). The B spectrum showed the lowest value (0.11 g),^4^ highlighting that red light can favor biomass accumulation due to its role in photosynthetic metabolism.

Figure 2 shows the development of the pepper plants under different spectra.

**Figure 2:**
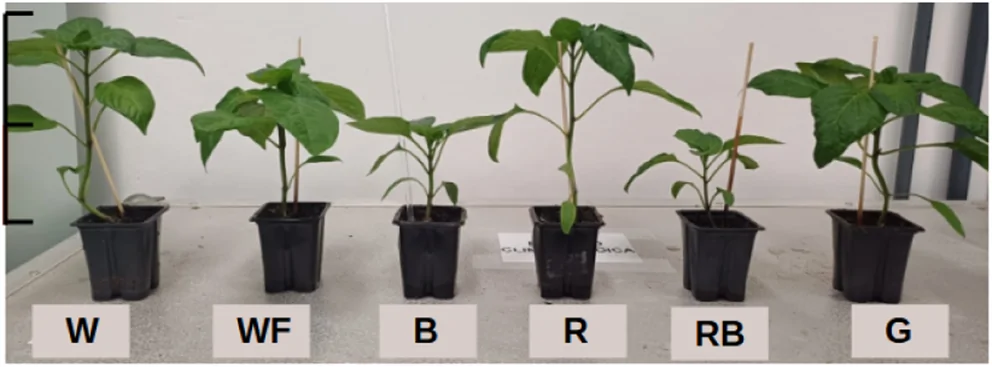
Bell pepper plants grown under different light spectra. The scale is divided into two parts; each part corresponds to 7.5 cm. White light (W), full-spectrum white light (WF), blue (B – 450 nm), red (R – 660 nm), 50% red (660 nm) and 50% blue (450 nm) (RB), and green (G – 530 nm).

The evaluated biometric parameters showed that, in the presence of R and WF light, the plants presented the most balanced architecture with greater leaf area and production of root and shoot biomass. When W light was used, the plants showed a greater internode distance and a larger leaf area. G light promoted plants with balanced shoot biomass and root length, while plants exposed to B light showed the lowest performance in the biometric parameters among the evaluated spectra.

Although the RB treatment produced more compact plants, this behavior should not be interpreted as being indicative of lower physiological quality. As demonstrated later in the nutritional analysis (Table 5), this spectrum promoted the greatest accumulation of Zn, Cu, and Mn, which are micronutrients directly involved in the activity of antioxidant enzymes, electron transport, and metabolic processes related to plant growth.

Thus, the lower height observed under RB may represent a photomorphogenic response resulting from the combined action of red and blue light on phytochromes, cryptochromes, and phototropins, favoring the development of more compact, yet metabolically active seedlings with high physiological potential, a characteristic considered desirable for seedlings intended for transplanting.

The different results presented, in the biometric parameters, especially for the R and B spectra (660 and 450 nm, respectively), are associated with the differences in the quantum efficiencies of these two wavelengths, which provide distinct photoelectric conversions and, when combined, result in varying energy consumption by the plant.^4^

The performance of pepper plants for the photosynthetic parameters evaluated in the different treatments is presented in Table 3.

**Table 3:** Performance of Bell Pepper Plants for the Photosynthetic Parameters Evaluated in the Different Treatments

Treatment	Instantaneous chlorophyll fluorescence^a^ (FT)	Quantum efficiency of PS II (QY)	Transpiration rate (E) (μmol_H2O_m^–2^s^–1^)	Stomatal conductance^a^ **-(**g) (molm^–2^s^–1^)	Photosynthetic assimilation rate (A) (μmol_CO2_m^–2^s^–1^)	Water use efficiency (USA) (μmol_CO2_mmol _H2O_)	Instantaneous carboxylation efficiency^a^ (EiC) (μmol m^–2^s^–1^(μmol mol^–1^)^−1^)
W	3876.66 ± 179.54	0.81 ± 0.01 a	1.01 ± 0.37 abc	0.07 ± 0.03	7.25 ± 1.41 a	7.63 ± 1.72 b	0.02 ± 0.00
WF	3860.00 ± 155.88	0.79 ± 0.01 ab	0.90 ± 0.09 abc	0.05 ± 0.01	5.65 ±1.01 abc	6.23 ± 0.81 b	0.02 ± 0.00
B	3996.67 ± 322.27	0.79 ± 0.01 ab	1.75 ± 0.55 a	0.06 ± 0.04	3.13 ± 0.26 c	1.93 ± 0.76 c	0.01 ± 0.00
R	3698.33 ± 204.35	0.81 ± 0.01 a	0.38 ± 0.08 c	0.03 ± 0.01	4.59 ± 1.48 abc	11.79 ± 1.40 a	0.02 ± 0.01
RB	3544.00 ± 341.74	0.78 ± 0.01 b	0.75 ± 0.21 bc	0.04 ± 0.01	4.02 ± 0.24 bc	5.75 ± 1.25 b	0.01 ± 0.00
G	3569.33 ± 139.41	0.76 ± 0.01 c	1.29 ± 0.30 ab	0.08 ± 0.03	6.33 ± 0.89 ab	5.00 ± 0.50 bc	0.02 ± 0.00
p-value	0.1935	0.0002	0.0033	0.5627	0.0029	<0.0001	0.0788
Coefficient of variation (%)	6.32	1.07	30.96	40.90	19.48	18.03	28.54

a Not significant by ANOVA at 5% probability of error. Values presented as mean ± standard deviation. White light (W), full-spectrum white light (WF), blue (B – 450 nm), red (R – 660 nm), 50% red (660 nm) and 50% blue (450 nm) (RB), and green (G – 530 nm).

Considering the results presented for the FT, it is possible to verify that the treatments did not differ statistically from each other, indicating that the amount of energy absorbed by the photosystems in the presence of the light source was similar and that there was no impairment of the light absorption capacity of the photosynthetic apparatus of the plants.

For QY, it was observed that plants subjected to W light and R light showed greater PSII efficiency (0.81), not differing statistically from WF and B, which presented a QY of 0.79. G light differed from the other treatments, showing a reduced value of 0.76, which may be associated with greater dissipation of nonphotochemical energy as a response to light stress. This energy dissipated in the form of heat and fluorescence in the PSII reaction centers may imply the inability of plants to protect themselves from damage caused by excess light.^33^

The reduction in photosystem II (PSII) quantum efficiency observed under the G spectrum (Table 4) may be associated with an energy imbalance in the photosynthetic apparatus, resulting from the way that G radiation is absorbed and distributed in the leaf mesophyll. Unlike B and R radiation, G light has a greater capacity to penetrate leaf tissues, which may result in a less uniform distribution of energy between reaction centers, promoting a state of physiological adjustment that does not necessarily optimize the photochemical efficiency of PSII.^33^

**Table 4:** Biochemical Characteristics of Pepper Plants Produced under Different Light Spectra

Treatment	Chlorophyll A^a^ (g kg^–1^)	Chlorophyll B^a^ (g kg^–1^)	Total chlorophyll^a^ (g kg^–1^)	Total flavonoids^a^ (mg 100 g^–1^)	Total phenolics (mg 100 g^–1^)
W	0.11 ± 0.02	0.20 ± 0.04	0.31 ± 0.05	232.20 ± 36.34	285.32 ± 48.89 b
WF	0.11 ± 0.02	0.24 ± 0.04	0.35 ± 0.07	189.98 ± 26.87	320.67 ± 12.60 b
B	0.11 ± 0.00	0.21 ± 0.02	0.32 ± 0.02	205.18 ± 12.99	275.29 ± 39.73 b
R	0.09 ± 0.01	0.17 ± 0.02	0.26 ± 0.03	208.91 ± 55.79	344.33 ± 12.98 b
RB	0.12 ± 0.01	0.23 ± 0.02	0.35 ± 0.01	244.16 ± 36.03	306.34 ± 27.04 b
G	0.10 ± 0.00	0.20 ± 0.01	0.30 ± 0.01	203.99 ± 17.29	482.47 ± 79.21 a
p-value	0.1579	0.1145	0.1145	0.4325	0.0009
Coefficient of variation (%)	12.22	13.43	12.40	15.86	12.92

a Not significant by ANOVA at 5% probability of error. Values presented as mean ± standard deviation. White light (W), full-spectrum white light (WF), blue (B – 450 nm), red (R – 660 nm), 50% red (660 nm) and 50% blue (450 nm) (RB), and green (G – 530 nm).

In this context, part of the absorbed light energy can be directed to nonphotochemical dissipation mechanisms, acting as a protection strategy against possible energy excesses.^26^ This process reduces the fraction of energy effectively converted into electronic transport, contributing to the decrease in quantum efficiency observed under G light.^26^ Thus, the reduction in QY may reflect more of a photoprotective adjustment mechanism than actual structural damage to the photosystem II.

In parallel, the significant increase in phenolic compounds under the G spectrum may suggest a functional integration between secondary metabolism and photoprotection processes.^26^ Phenolic compounds, especially flavonoids, act as important regulators of the cellular redox state, contributing to the neutralization of reactive oxygen species and the stabilization of oxidative balance.^38^ The induction of the phenylpropanoid pathway under G light may, therefore, represent a metabolic response associated with the control of oxidative stress generated by the energy adjustment of PSII.^26,37^

Thus, it is observed that the reduction in PSII efficiency under G light is closely related to a physiological programming, in which part of the absorbed energy is dissipated through nonphotochemical dissipation mechanisms that concomitantly direct metabolism toward the synthesis of phenolic compounds.^26,38^ This integrated adjustment suggests that G light acts not only as a modulator of photosynthesis but also as a regulatory signal of secondary metabolism and photoprotection mechanisms, reinforcing the interaction between photochemical efficiency and antioxidant response in pepper seedlings.

Stomatal conductance did not differ statistically between the treatments. In the study by,^31^ when G light (524 nm) was included in the spectrum, tomato plants showed a reduction in stomatal conductance and transpiration rate. This response of the G spectrum to the reduction in transpiration rate was not observed in this study and may be associated with some alteration in PSII that promoted better functioning of the photosynthetic apparatus,^33^ in addition to the genetic factor associated with the species.

The highest photosynthetic assimilation occurred in light W (7.25 μmol_CO_2__ m^–2^ s^–1^), which favored plant growth. Lights WF, R, and G did not differ statistically from light W, highlighting the photosynthetic performance associated with greater efficiency in enzymatic processes. Light B showed the lowest result among the treatments (3.13 μmol_CO_2__ m^–2^ s^–1^), possibly indicating the low efficiency of using this spectral range in carbon assimilation. Generally, this wavelength is considered photosynthetically less efficient,^31^ therefore, although light B stimulates processes related to stomatal opening, its isolated use may limit the overall efficiency of carbon fixation by not providing the spectral balance necessary to maximize electron transport and net assimilation.

In an integrated manner, it is observed that the highest photosynthetic assimilation rates recorded under the W, WF, and R spectra (Table 4) coincide with the accumulation of shoot dry biomass (Table 2) for the same treatments. This behavior suggests that the increase in biomass may be associated with greater efficiency in carbon fixation, indicating that the spectral quality favored the conversion of photoassimilates to plant growth. This relationship reinforces the hypothesis that net photosynthetic assimilation may play an important role in the accumulation of dry matter, especially under spectra with a greater contribution in the red range and a balanced spectrum.^8^

The better relationship between CO_2_ fixation and water losses in the system was observed in color R when analyzing water use efficiency (11.79 μmol_CO2_ mmol_H2O_^–1^), due to the lower transpiration rate. In contrast, light B showed the lowest water use efficiency among the treatments (1.93 μmol_CO2_ mmol_H2O_^–1^), with the highest transpiration rate.

The greater water-use efficiency observed under the R spectrum (Table 4) suggests a physiological growth strategy associated with optimized carbon fixation with lower water losses. However, this physiological pattern may be related to more elongated growth, potentially characteristic of photomorphogenic responses induced by red, which indicates that, although physiological efficiency is high, adjustments to the spectrum may be necessary to avoid excessive etiolation in seedling production systems.^23^

The instantaneous carboxylation efficiency showed no statistical difference between treatments, demonstrating that, regardless of the spectrum used, the Rubisco enzyme activity remained constant, because the carboxylation efficiency related to CO_2_ flow is associated with stomatal conductance.^31^

Yousef et al.^33^ pointed out that spectral combination can favor the performance of the photosynthetic apparatus. However, in the present study, no better performance of the RB spectrum (440 nm/670 nm) was observed in relation to the quantum efficiency of PSII, transpiration rate, photosynthetic assimilation rate, and water use efficiency for the aforementioned spectral combination.

Soufi et al.,^30^ when evaluating two lettuce varieties (Lollo Rossa and Lollo Bionda), observed that the use of LEDs benefited photosynthetic parameters, such as net photosynthesis rate, stomatal conductance, transpiration rate, CO_2_ fixation in leaves, and water use efficiency, thus increasing the efficiency of the photosynthetic apparatus.

The results obtained for the biochemical parameters produced by the plants as a function of exposure to different light spectra are presented in Table 4.

The parameters of chlorophyll A, B, and total chlorophyll did not show a significant difference among treatments. Only the total phenolics differed with a higher value in the G spectrum. According to ref. 14, LEDs promote high efficiency in the emission of PAR radiation, which may justify the absence of significant differences between treatments. In addition, the high physiological plasticity of the seedlings may contribute to the stability of the photosynthetic apparatus.

Nie et al.^23^ observed a reduction in chlorophyll under red light and an increase under blue light, unlike what was observed in this study. The differences observed between studies may be related to the light intensity, exposure time, and physiological characteristics of the species. In addition, photosynthetic acclimation mechanisms may maintain stable chlorophyll levels, even under different spectra, reducing phenotypic variations.

From observing Table 3, it is possible to verify that the values obtained for the total flavonoid content did not differ statistically from each other. When evaluating different species of vegetables, Maran et al.^20^ were able to observe that supplementation with artificial light W (40% red – 670 nm +10% blue – 430 nm +50% green – 530 nm) and the RB combination (87.5% red – 670 nm +12.5% blue – 430 nm) did not affect the production of phenolic compounds and flavonoids for red cabbage and mustard crops. In contrast, Rizzon et al.^26^ found higher levels of phenolic compounds and flavonoids in lettuce plants supplemented with RB light (red: 670 nm and blue: 430 nm). It is important to note the distinct behavior of plant species for the different wavelengths, as mentioned by Lazzarini et al.^18^

The statistically higher content of phenolic compounds for the G spectrum compared to the other light spectra suggests a modulation of secondary metabolism in response to light quality. Although the effects of B and R light are widely documented, recent studies demonstrate that G light also acts as an important regulatory signal for physiological and metabolic processes, influencing the biosynthesis of secondary metabolites by altering gene expression and enzymatic activity associated with bioactive compound biosynthetic pathways.^26,38,44^

Activation of the phenylpropanoid pathway, mediated by the PAL enzyme, may explain this behavior.^36–38^ Furthermore, greater penetration of green light into leaf tissues may intensify metabolic acclimation responses.^31,44^ Phenolics also act as antioxidants, protecting against reactive oxygen species (ROS).^38^ Additionally, they exert a photoprotective function by dissipating excess light energy.^37^ Therefore, the higher levels of phenolic compounds under green light can be attributed to the integration among the metabolic activation of the phenylpropanoid pathway, antioxidant response, and photoprotective mechanisms, reinforcing the role of spectral quality in the physiological and biochemical modulation of plants.

Flavonoids and other derivatives of the phenylpropanoid pathway act in the absorption of part of the incident radiation and in the dissipation of excess light energy, reducing the occurrence of photo-oxidation and protecting sensitive chloroplast structures, especially photosystems.^37^ Thus, the increase in phenol levels under green light may represent a photoprotective response aimed at preserving the integrity of the photosynthetic apparatus and optimizing the use of available light energy.^37^

Therefore, the higher levels of phenolic compounds observed under the green spectrum can be attributed to the combined action of three complementary mechanisms: (i) stimulation of the phenylpropanoid pathway and PAL activity;^36,38^ (ii) strengthening of antioxidant capacity in response to ROS signaling;^38^ and (iii) induction of photoprotective and adaptive mechanisms related to acclimation to the light environment.^37^ These results reinforce that the spectral quality of light acts not only on seedling growth but also on secondary metabolism, directly influencing the synthesis of bioactive molecules associated with cell protection and physiological adaptation of plants.

Regarding nutrient absorption by bell pepper plants, the results for the levels of macronutrients present in the plant tissues for the different treatments are presented in Table 5.

**Table 5:** Macronutrient Content in the Plant Material of Bell Pepper Plants in the Different Treatments

Treatment	Nitrogen^a^ (g·kg^–1^)	Phosphorus^a^ (g·kg^–1^)	Potassium (g·kg^–1^)	Calcium (g·kg^–1^)	Magnesium^a^ (g·kg^–1^)	Sulfur (g·kg^–1^)
W	40.83 ± 5.22	8.50 ± 0.46	48.33 ± 13.57 b	8.83 ± 0.89 ab	9.20 ± 0.69	4.37 ± 0.32 ab
WF	48.57 ± 3.27	11.67 ± 0.12	72.37 ± 3.67 a	8.17 ± 0.40 b	9.83 ± 0.46	4.20 ± 0.44 ab
B	53.07 ± 3.98	12.00 ± 0.96	68.50 ± 4.77 a	8.73 ± 0.40 ab	10.20 ± 0.44	4.73 ± 0.32 a
R	45.03 ± 7.25	9.20 ± 1.65	80.37 ± 5.43 a	8.37 ± 0.58 ab	9.97 ± 0.84	4.30 ± 0.53 ab
RB	53.15 ± 1.95	12.40 ± 0.81	66.37 ± 6.27 ab	9.97 ± 0.58 a	10.60 ± 0.44	4.33 ± 0.12 ab
G	45.25 ± 5.45	10.17 ± 1.40	71.50 ± 2.65 a	7.80 ± 0.78 b	9.27 ± 1.36	3.53 ± 0.29 b
p-value	0.0515	0.0026	0.0028	0.0193	0.2751	0.0322
Coefficient of variation (%)	10.13	9.78	10.34	7.32	7.89	8.45

a Not significant by ANOVA at 5% probability of error. Values presented as mean ± standard deviation. White light (W), full-spectrum white light (WF), blue (B – 450 nm), red (R – 660 nm), 50% red (660 nm) and 50% blue (450 nm) (RB), and green (G – 530 nm).

Analysis of nutrient content revealed differences in absorption and nutrient accumulation under different light spectra. The content of N did not show significant differences between treatments, indicating that all spectra were able to induce the absorption of this nutrient in plant material with the same demand. Therefore, the availability of photosynthetic energy from the different spectra did not influence nitrogen metabolism, which is important for protein synthesis and vegetative growth.

For P, there were also no significant differences between treatments, demonstrating that like N, the absorption of this nutrient, essential for energy and photosynthetic processes, remained stable regardless of the light spectrum.

Similarly, the Mg content in the plant material of the bell pepper plants also did not show a statistically significant difference between them, with this element being related to the structural function in the chlorophyll molecule (which did not show a statistically significant difference between treatments as previously presented) and in essential enzymatic processes, regardless of the light spectrum.

The K levels showed statistical differences between them, with the lowest accumulation of the nutrient observed under W light (48.33 g·kg^–1^) and the highest level of the element under R light (80.37 g·kg^–1^), which did not differ statistically from the other spectra. This suggests that the evaluated spectra may favor the translocation and accumulation of K, a fundamental element for osmotic regulation, stomatal opening, and photosynthesis.^1,10^

The highest Ca content was observed in the RB light combination treatment (9.97 g·kg^–1^), differing from the lowest values under WF light (8.17 g·kg^–1^) and G light (7.80 g·kg^–1^). The variation may be related to physiological adjustments, since Ca plays an important role in cell signaling and membrane stability. This same trend of higher Ca absorption in the RB combination treatment was verified by Soufi et al.,^30^ using an RB light ratio of 3:1 and wavelengths of 656 and 450 nm, highlighting that the spectral combination showed a beneficial influence on nutrient absorption and, consequently, on the growth and development of two lettuce varieties (Lollo Rossa and Lollo Bionda).

Calcium plays fundamental structural and regulatory roles in plants, acting both in cell wall formation and in maintaining the stability of cell membranes.^1,19^ In addition, Ca^2+^ participates in intracellular signaling mechanisms, functioning as a secondary messenger in the regulation of physiological and metabolic responses associated with growth and environmental adaptation.^39,40^

Since Ca transport occurs mainly via transpiration flow, changes induced by the spectral quality of light can influence its absorption and distribution in tissues.^46^ From an agronomic point of view, seedlings with an adequate calcium supply tend to exhibit greater cell integrity, better establishment, and greater recovery capacity after transplanting.^22^

Regarding the S content in the plant material, the highest value was presented by light B (4.73 g·kg^–1^), differing statistically only from the levels recorded under light G (3.53 g·kg^–1^). OS is directly related to the synthesis of the sulfur-containing amino acids cysteine and methionine, which are essential for protein formation and plant metabolism.^19,21^

Cysteine also acts as a precursor to glutathione, one of the main cellular antioxidants, responsible for maintaining redox homeostasis and protection against reactive oxygen species (ROS).^3^ Thus, variations in S levels may reflect physiological adjustments related to the antioxidant capacity of plants, especially under different light conditions, contributing to metabolic protection and adaptation to their growing environment.

The behavior observed for the macronutrients N, P, K, and Ca in this study aligns with the observations of ref. 12, where different mint species presented similar levels of N, P, and K but differed in relation to Ca levels. Regarding Mg content, the result presented in the present study differed from that observed by.^12^

Rizzon et al.^26^ observed, in a study with vegetables (lettuce and cauliflower), that seedlings grown with supplemental lighting can produce a greater amount of biomass and a larger leaf area through the optimization of the use of photoassimilates, enhancing the effect of different wavelengths, and thus may present reduced nutritional levels in the plant material, since the plants drain them for metabolic activities.

Thus, regardless of the spectrum used, the elements N, P, K, and Mg showed less sensitivity for absorption and nutrient assimilation by pepper plants than did the macronutrients Ca and S, which were more sensitive to the changes caused by the spectra studied.

The results obtained in the evaluation of micronutrients present in the plant material of pepper plants subjected to different spectra are presented in Table 6.

**Table 6:** Micronutrient Content in the Plant Material of Pepper Plants under Different Treatments

Treatment	Zinc (mg·kg^–1^)	Copper (mg·kg^–1^)	Manganese (mg·kg^–1^)	Iron (mg·kg^–1^)	Boron^a^ (mg·kg^–1^)
W	66.13 ± 2.38 d	7.67 ± 0.31 d	49.87 ± 4.01 bc	142.33 ± 30.56 b	46.97 ± 8.64
WF	72.17 ± 1.20 cd	8.80 ± 0.62 cd	52.00 ± 2.38 b	159.53 ± 21.95 ab	36.17 ± 7.36
G	84.53 ± 1.57 b	12.97 ± 0.23 b	64.67 ± 0.45 ab	193.70 ± 16.11 a	45.33 ± 14.18
R	76.27 ± 4.65 c	9.77 ± 0.25 c	58.33 ± 3.03 b	167.37 ± 8.07 ab	38.80 ± 12.64
RB	92.60 ± 1.73 a	15.07 ± 0.75 a	75.37 ± 4.18 a	176.50 ± 3.41 ab	56.67 ± 16.25
B	73.85 ± 3.55 c	10.23 ± 0.72 c	34.80 ± 11.57 c	139.30 ± 14.13 b	54.40 ± 0.10
p-value	<0.0001	<0.0001	<0.0001	0.0237	0.2358
Coefficient of variation (%)	3.59	4.93	9.87	11.05	24.16

a Not significant by ANOVA at 5% probability of error. Values presented as mean ± standard deviation. White light (W), full-spectrum white light (WF), blue (B – 450 nm), red (R – 660 nm), 50% red (660 nm) and 50% blue (450 nm) (RB), and green (G – 530 nm).

Analysis of micronutrient levels revealed statistically significant differences among the Zn, Cu, Mn, and Fe treatments, indicating that different light conditions can directly influence the absorption and translocation of these elements. However, no significant changes were observed for element B regardless of the spectrum used.

The Zn content showed the highest value in the treatment with the RB spectrum combination (92.60 mg·kg^–1^), differing statistically from all others. This may indicate a synergistic effect of the RB spectrum on the metabolic processes associated with Zn absorption, which is an essential element for auxin synthesis and antioxidant enzyme activity.^3^ The lowest efficiency in Zn absorption was observed in the W light (66.13 mg·kg^–1^).

Regarding Cu absorption by pepper plants, the highest levels were recorded in the RB light combination (15.07 mg·kg^–1^), while W light showed the lowest element content, 7.67 mg·kg^–1^. Cu is a fundamental enzymatic cofactor for oxidative and photosynthetic metabolism, and these results suggest that the RB light combination may favor the activity of transporters related to its assimilation.^3^

Mn is an essential element for the photolysis of water and electron transport in photosystem II. Mn deficiencies can impair the light response during photosynthesis due to deficiencies in electron transport.^3^ The highest Mn content in the plant material was recorded in the RB light combination treatment (75.37 mg·kg^–1^). In contrast, only light G showed the lowest Mn content (34.80 mg·kg^–1^), indicating a possible limiting effect of this spectrum on Mn absorption or translocation, and the greater efficiency of the RB spectrum in favoring the absorption of this micronutrient.

The Fe levels present in the plant material varied from 139.30 mg·kg^–1^ in light G to 193.70 mg·kg^–1^ in light B, showing statistical differences. Light B, in this case, showed the possibility of enhancing the accumulation of this micronutrient, which is important for chlorophyll synthesis and electron transport processes, acting in the processes of photosynthesis and respiration.^3^

Soufi et al.^30^ observed, in lettuce plants (Lollo Rossa and Lollo Bionda), that the RB spectral combination allowed for a greater accumulation of the micronutrients Cu, Zn, Mn, and Fe in the plant material. This same trend was presented by the pepper plants in the present study. Although the highest levels of Mn and Fe were observed under the RB and B spectra (Table 6), these nutritional increments were not directly reflected in proportional increases in the photosynthetic efficiency or in the quantum efficiency of PSII (Table 3). This result suggests that, despite the greater availability of these micronutrients essential for the functioning of the photosynthetic apparatus, other physiological or regulatory factors may have limited photochemical performance, reinforcing that nutritional absorption does not necessarily imply a direct photosynthetic response.^2,28^

Marks et al.^12^ observed significant differences between the different spectra evaluated in mint plants for the micronutrients Cu, Zn, Mn, and Fe, attributing these results to possible genetic characteristics and the impact of light on plant growth and development, which agrees with the results obtained in this study.

Among the micronutrients, B did not show statistical differences in the plant material. It is worth highlighting that it is an essential micronutrient for cell growth and differentiation and sugar transport,^3^ which may possibly not exhibit absorption sensitivity in relation to the different light frequencies used.

Kizil et al.^15^ highlight that the influence of light stimulus can lead to changes in the way nutrients are absorbed by plants, as it interferes with photoreceptors, triggering signals that promote biochemical changes and even gene transcription in plants, thus promoting distinct behaviors between different spectra, as observed in the present study.

Thus, it was observed that the RB spectral combination promoted greater absorption of Zn, Cu, Mn, and Fe, possibly creating a more favorable environment for the metabolism and absorption of these micronutrients. This light combination proved to be beneficial in optimizing the nutritional and physiological performance of pepper plants, as it may have promoted increased expression of root transporters and metabolic activity associated with nutrient absorption, justifying the greater accumulation of Zn, Cu, and Mn observed.

The greatest accumulation of micronutrients under the RB spectrum occurred even though this treatment did not result in greater height or a higher rate of photosynthetic assimilation. This behavior suggests that the response promoted by this spectral combination may be more associated with improving the physiological quality of the seedlings than with simply stimulating plant elongation.

In seedling production systems, more compact plants exhibit less susceptibility to etiolation, greater structural robustness, and better performance after transplanting, making the RB combination particularly interesting for production programs in controlled environments. Thus, seedlings with higher micronutrient contents may show better establishment after transplanting, greater initial photosynthetic capacity, and greater tolerance to biotic and abiotic stresses.

In general, nutritional enrichment directly influences photosynthesis by modifying the availability of metabolic substrates and the efficiency of the biochemical reactions associated with carbon metabolism. However, this relationship is not exclusively linear, since biochemical limitation can occur in which the enzymatic capacity of the photosynthetic system becomes a limiting factor, even under adequate nutritional supply conditions.

Furthermore, the plant’s metabolic response is dynamically regulated, involving metabolic regulatory mechanisms that adjust the flow of assimilates according to physiological demand and environmental conditions. This adjustment can result in a decoupling between nutritional status and instantaneous photosynthetic performance, especially in systems under varying light conditions, where photosynthetic efficiency may not immediately reflect the plant’s nutritional status.

Figure 3 presents the principal component analysis (PCA) of the biometric, photosynthetic, biochemical, and nutritional parameters of pepper seedlings grown under different LED light spectra.

**Figure 3:**
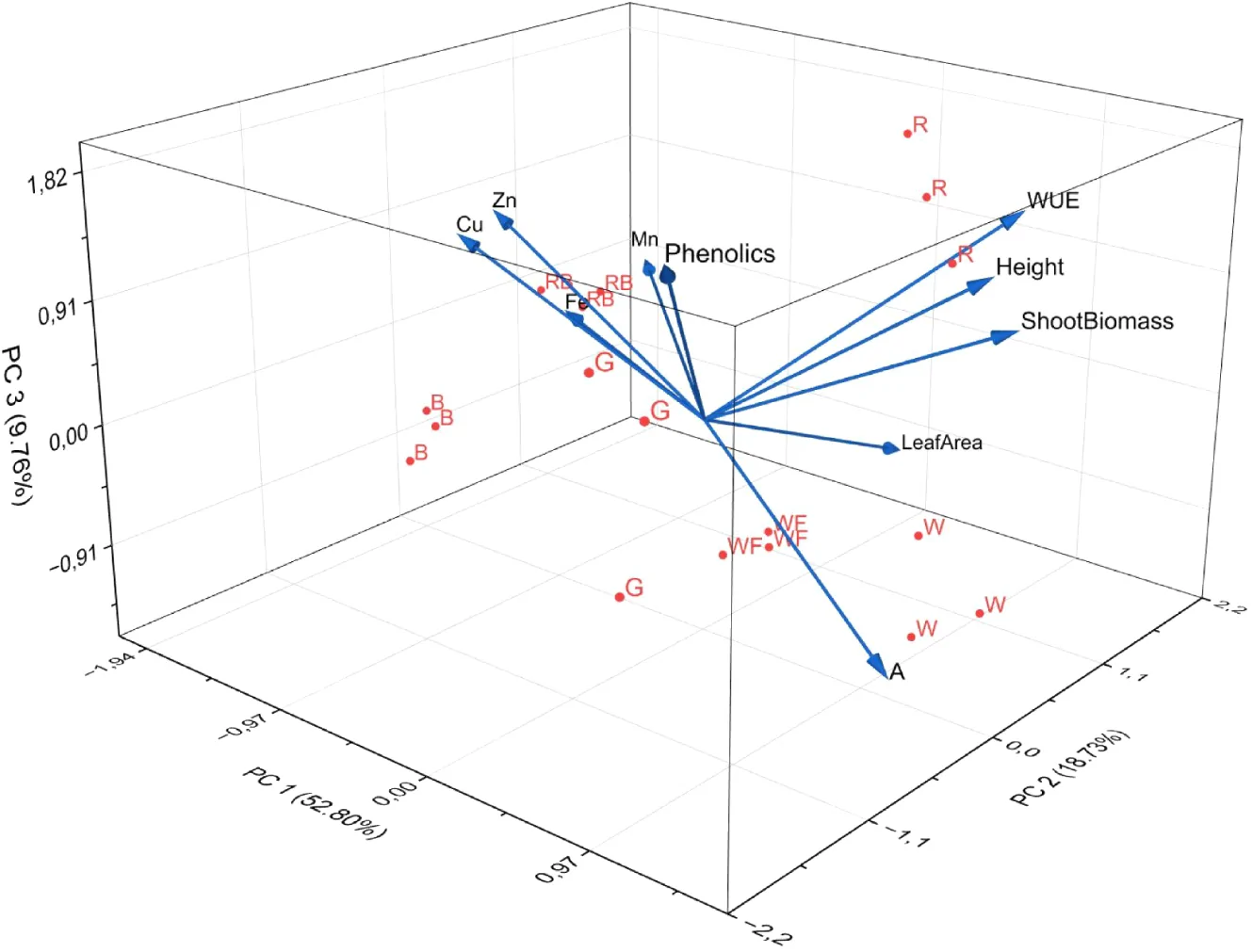
Principal component analysis (PCA) of biometric, photosynthetic, biochemical, and nutritional parameters of pepper seedlings grown under different LED light spectra: white light (W), full-spectrum white light (WF), blue (B – 450 nm), red (R – 660 nm), 50% red (660 nm) and 50% blue (450 nm) (RB), and green (G – 530 nm).

Principal component analysis (PCA) allowed for an integrated interpretation of the biometric, physiological, biochemical, and nutritional responses of pepper seedlings subjected to different light spectra (Figure 3). This is because the first three principal components explained 80.46% of the total variability of the data, with PC1 representing 52.80%, PC2 representing 18.13%, and PC3 representing 9.76%, demonstrating a satisfactory representation of the experimental variability.

Seedlings grown under red light showed a positive association with plant height, shoot biomass accumulation, and water-use efficiency (WUE), indicating that red light may have favored carbon allocation for vegetative growth and improved physiological efficiency. This behavior corroborates the biometric and photosynthetic results discussed previously, reinforcing the role of red wavelengths in promoting the elongation growth and biomass accumulation.

The RB spectral combination showed a strong association with the accumulation of micronutrients, such as Zn, Cu, and Fe, indicating that the interaction between red and blue wavelengths may stimulate nutrient absorption and translocation mechanisms. This result may strengthen the hypothesis that combined spectra optimize metabolic and nutritional processes more efficiently than do monochromatic light.

The G spectrum showed a stronger association with total phenolic compounds, suggesting the activation of the secondary metabolism and antioxidant defense mechanisms. This response is consistent with the observed reduction in the quantum efficiency of PSII under green light, indicating that this spectrum may induce moderate physiological stress and stimulate the protective metabolic pathways.

The W and WF spectra were positioned closer to the photosynthetic assimilation rate and leaf area, indicating a more balanced physiological performance and a greater photosynthetic efficiency. According to the data, these spectra may help promote more homogeneous seedling development, suggesting their potential use in commercial seedling production systems that are aimed at balanced growth and physiological stability.

In contrast, spectrum B showed a weaker association between variables related to growth and physiological efficiency, corroborating the lower biometric performance and photosynthetic assimilation observed at this wavelength. This behavior suggests that monochromatic blue light, under the evaluated growing conditions, may not be sufficient to maximize the initial development of the pepper seedlings.

In general, PCA showed that different light spectra promote distinct physiological and metabolic responses in pepper seedlings, allowing for the identification of specific associations between treatments and the variables evaluated. These results reinforce the potential of spectral manipulation as a complementary tool for modulating characteristics related to growth, physiological performance, and seedling metabolism.

The results also suggest that light spectra can contribute differently to seedling development depending on the production objective. Red light was more related to vegetative growth and biomass accumulation, while the red-blue combination was associated with greater micronutrient accumulation and seedlings with more compact characteristics. White light spectra favored a more balanced physiological performance, while green light was associated with an increased synthesis of phenolic compounds, indicating possible stimulation of antioxidant metabolism and the production of bioactive compounds.

It is important to note that the experiment was conducted using a single pepper cultivar under controlled environmental conditions and a fixed daily light integral (DLI), which may limit the extrapolation of the results to other production systems or genotypes. Furthermore, the study focuses on the seedling stage; that is, posttransplant establishment of seedlings under field or greenhouse conditions was not verified. Therefore, further studies are needed to validate the long-term effects of using different light spectra on the crop performance.

## Conclusion

The results demonstrate that the spectral quality of light is a determining factor in modulating the initial development of pepper seedlings, influencing growth, photosynthetic performance, secondary metabolism, and nutrient absorption in an integrated manner. The combined analysis of the variables, corroborated by principal component analysis, showed that each spectrum can induce specific physiological responses, and there is no standard light condition for all of the parameters evaluated. Thus, the choice of the light spectrum tends to be guided by the objective of seedling production. While some spectra favor growth and photosynthetic efficiency, others can enhance nutritional enrichment or stimulate metabolic protection mechanisms, demonstrating that manipulating the light quality can be used as a strategy to target physiological and agronomic attributes of interest. These findings broaden our understanding of the mechanisms by which different spectral compositions can regulate the development of pepper seedlings and reinforce the potential of LED technology as a tool to optimize production systems in controlled environments, contributing to greater efficiency and sustainability in nurseries and indoor cultivation systems.
