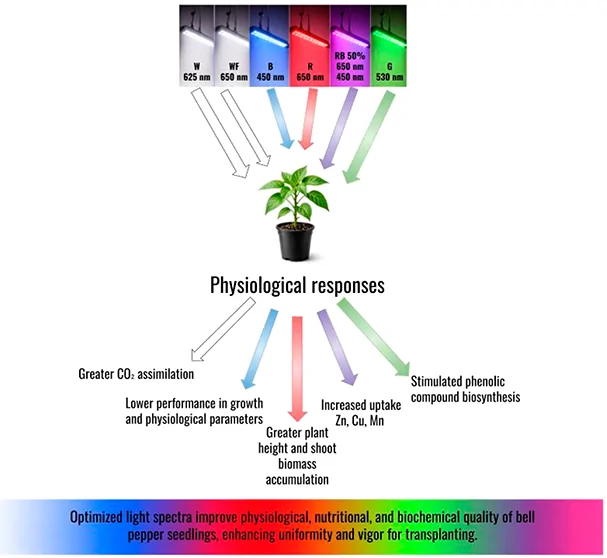# Correction to “Influence of Different Light Spectra on Metabolism, Nutrition, and Photosynthetic Efficiency in the Development of Bell Pepper Seedlings”

**DOI:** 10.1021/acsomega.6c09775

**Published:** 2026-10-05

**Authors:** Vanessa Susana Rech Bisi, Henrique Poersch, Ketelyn Rubert, Ruan Fogaça Feijó, Wendel Paulo Silvestre, Gabriel Fernandes Pauletti

The original TOC graphic was incorrectly taken from another paper (10.1021/acsomega.6c03840). The correct TOC graphic is provided below.